# Continuous controversy about radiation oncologists’ choice of treatment regimens for bone metastases: should we blame doctors, cancer-related features, or design of previous clinical studies?

**DOI:** 10.1186/1748-717X-8-85

**Published:** 2013-04-10

**Authors:** Carsten Nieder, Adam Pawinski, Astrid Dalhaug

**Affiliations:** 1 Department of Oncology and Palliative Medicine, Nordland Hospital, Bodø, 8092, Norway; 2 Institute of Clinical Medicine, Faculty of Health Sciences, University of Tromsø, Tromsø, 9038, Norway

**Keywords:** Radiotherapy, Radiation oncology, Bone metastases, Fractionation regimen

## Abstract

Recent studies from Italy, Japan and Norway have confirmed previous reports, which found that a large variety of palliative radiotherapy regimens are used for painful bone metastases. Routine use of single fraction treatment might or might not be the preferred institutional approach. It is not entirely clear why inter-physician and inter-institution differences continue to persist despite numerous randomized trials, meta-analyses and guidelines, which recommend against more costly and inconvenient multi-fraction regimens delivering total doses of 30 Gy or more in a large number of clinical scenarios. In the present mini-review we discuss the questions of whether doctors are ignoring evidence-based medicine or whether we need additional studies targeting specifically those patient populations where recent surveys identified inconsistent treatment recommendations, e.g. because of challenging disease extent. We identify open questions and provide research suggestions, which might contribute to making radiation oncology practitioners more confident in selecting the right treatment for the right patient.

## Introduction

Most radiotherapy facilities worldwide are treating large numbers of patients with bone metastases from solid tumors. Over the last decades, numerous prospective randomized trials have confirmed the high efficacy of radiation treatment in terms of pain relief. The presence of pain does not seem to be correlated with the type of tumor, location, number or size of the metastases [1,2]. For localized pain, radiotherapy is a well-accepted treatment modality with a 60-80% likelihood of overall pain relief reported [3-5]. The mechanism of response remains unknown. Because the onset of pain relief is often rapid, within days, it is not likely to be dependent upon tumor shrinkage alone. It is probable that a response mechanism through modification of chemical mediators is important.

Different palliative radiotherapy schedules have been studied, ranging from a single fraction of 6 or 8 Gy, to 20 Gy in 5 fractions, 24 Gy in 6 fractions, 30 Gy in 10 fractions and even 40 Gy in 20 fractions [6-22]. No clear dose effect relationship has been seen in any of these trials. Subsequent meta-analyses have confirmed the equal effectiveness of a single dose schedule compared to more protracted regimens [3,23,24]. Based on acute toxicity rates, convenience and cost/benefit ratio, a single fraction of 8 Gy is therefore considered to be the preferred radiotherapy schedule for patients with uncomplicated bone pain, that is for bone lesions not causing neurological complaints and without a high risk of pathological fracture. Among the randomized trials comparing single versus multiple fractions for painful bone metastases, retreatment rates were consistently higher after the single dose schedules. Percentages varied from 11-42% after a single fraction to 0-24% after multiple fractions.

If complicated by spinal canal invasion or compression of the spinal cord, common practice in most western countries is to deliver 20 Gy in 5 fractions in patients with a minimum life expectancy of 3 months, or 30 Gy in 10 fractions in patients with a more prolonged life expectancy (minimum 1 year) [25-28]. Single fraction treatment might be considered in patients with a prognosis less than 3 months.

### Recent data on utilization of single fraction radiotherapy

In Japan, members of the Japanese Radiation Oncology Study Group (JROSG) were invited to complete an Internet-based survey and describe the radiotherapy dose/fractionation they would recommend for 4 hypothetical cases [29]. Case 1 described a patient with an uncomplicated painful bone metastasis in a non-weight-bearing site from non-small-cell lung cancer. Case 2 investigated whether management for a case of uncomplicated spinal metastasis would be different from that in case 1. Case 3 was identical with case 2 except for the presence of neuropathic pain. Case 4 investigated the prescription for an uncomplicated painful bone metastasis secondary to oligometastatic breast cancer. A total of 52 radiation oncologists from 50 institutions responded. In all four cases, the most commonly prescribed regimen was 30 Gy in 10 fractions. Single fraction irradiation was recommended by 13% of respondents for case 1, 6% for case 2, 0% for case 3, and 2% for case 4.

A comparable survey was distributed in Italy to determine the decision patterns of Italian radiation oncologists in 4 different clinical cases of patients with bone metastases [30]. Again, the cases were different with respect to the histology of the primary tumor (breast, prostate and lung cancer), and in addition performance status, pain before and after analgesics, tumor site, and radiological characteristics of the metastatic lesions. One hundred twenty-two questionnaires were adequately completed and considered for the analysis. Single fraction radiotherapy was the preferred option in a minority of respondents in each case (ranging from 9-30%). Major factors influencing choice of dose/fractionation regimen included prognosis, performance status, metastatic site and radiological appearance of the lesion. Financial aspects, personal habits and departmental waiting lists were not among these factors (<7% of responses for each factor).

A different Internet-based survey was distributed to the members of 3 radiation oncology professional organizations (American Society for Radiology Oncology [ASTRO], Canadian Association of Radiation Oncology [CARO], Royal Australian and New Zealand College of Radiologists) [31]. It included 5 hypothetical patients with single or multiple painful metastases from breast, lung, or prostate cancer, plus two reirradiation scenarios. A total of 962 respondents, three-quarters ASTRO members, described 101 different dose schedules in common use. The median dose overall was 30 Gy in 10 fractions. Single fraction schedules were used the least often by ASTRO members practicing in the United States (3-16%) and most often by CARO members (17%, 31%, 38%, 56% and 69% in the 5 cases). Case, membership affiliation, country of training, location of practice, and practice type were independently predictive of the use of single fraction radiotherapy. The principal factors considered when prescribing were prognosis, risk of spinal cord compression, and performance status.

French speaking physicians from different European countries (n=644) also answered a questionnaire on bone metastases management [32]. Only 54% used short course radiotherapy in routine. Of 35 African centers contacted in a different study, 24 (68%) completed the questionnaire [33]. Fourteen centers had a single fractionation regimen as an institutional policy for treating painful bone metastases (one size fits all approach), whereof 5 centers (21%) used 8 Gy single fraction. Further surveys from different time periods and countries exist but these are not necessary to describe clinical practice because results were comparable to those already reviewed.

A different approach was taken in Norway, where a national registry-based study was conducted, including all radiotherapy schedules of 8 Gy single dose and 30 Gy in 10 fractions (1997–2007) [34]. A total of 14,380 radiotherapy courses were identified. During the time period 31% of the treatments were delivered as single fraction. The proportion of single fraction treatments increased from 16% in 1997 to 41% in 2007. There were substantial differences in the proportion of single fraction treatments between the treatment centers (range 25-54%). These differences persisted after adjustment for sex, age, primary diagnosis, anatomical region, and travel distance.

In a Canadian study, electronic records from the nine provincial radiotherapy centers in Ontario were linked to the Ontario Cancer Registry to identify all courses of palliative radiotherapy for bone metastases [35]. Between 1984 and 2001, 44,884 patients received 74,432 courses. The mean number of fractions per course was 3.9. The proportion of patients treated with a single fraction increased from 27% in 1984–1986 to 40% in 1987–1992 and decreased thereafter. Single fractions were used more frequently in patients with a shorter life expectancy, in older patients, and in patients who lived further from a radiotherapy facility. Single fractions were used more frequently when the prevailing waiting time was longer.

## Discussion

As shown above [29-31], the use of single fraction radiotherapy depends on patient- and disease-related factors. Moreover, considerable differences exist between countries and treatment facilities. The optimal utilization rate is unknown and depends on case mix, but could possibly be as high as the 54% reported as the highest rate in one Norwegian center. The Japanese and U.S. figures (<20%) were strikingly different and indicate under-utilization even if they are not related to actual patient treatments. What are the possible explanations for these variations in pattern of care? The landmark randomized trials and meta-analyses have been discussed and published extensively, including editorials and oral presentations at all national and international radiation oncology meetings. So, poor knowledge distribution and education are unlikely to explain the discrepancy between scientific evidence and clinical practice. Possibly, single fraction treatment does not fulfill some patients' expectation regarding high tech radiation oncology in a time of public discussion around protons, robotic radiosurgery and other developments. To improve this obstacle, better communication and patient information might be necessary. Previous studies suggested that most patients were not familiar with the concept of radiation treatment and that approximately 40% believed that palliative radiotherapy would prolong their life [36,37]. Up to 25% believed their cancer was curable.

Is it likely that variations in systemic cancer treatment influence fractionation concepts, need for, and efficacy of palliative radiotherapy for bone metastases? It has become common practice to administer bisphosphonates or denosumab, which reduce the likelihood of skeletal-related events (pathologic fracture, spinal cord compression, need for surgery and radiotherapy). At the same time, improved chemotherapy, endocrine treatment, and new targeted agents are able to extend survival of patients with metastastic cancer, thereby increasing the time period during which skeletal-related events might occur. The latter development might prevent radiotherapy utilization rates from declining, but the focus of this article is on fractionation. In theory, systemic therapy might act additive to radiotherapy and could replace a certain amount of dose while still maintaining the same level of efficacy. Moreover, initial use of systemic therapy might delay referral to radiotherapy and shift the patient population towards a heavier pretreated and maybe more recalcitrant state. The literature contains limited confirmatory information. A Japanese study of breast cancer patients with spinal column metastases suggested that local control and survival after radiotherapy were better in the absence of previous chemotherapy [38]. There is ample opportunity for prospective research projects on this subject because most previous studies provided little information about the interplay of systemic therapy and irradiation, and there is also rapid introduction of new drugs and regimens in diseases such as breast, prostate, kidney and bowel cancer. There is currently no high evidence data proofing that one particular fractionation regimen provides advantages over others in the most common scenarios, for example castration-resistant prostate cancer, endocrine responsive breast cancer, Her2 positive breast cancer, EGFR wild-type non-small cell lung cancer, clear cell kidney cancer treated with angiogenesis inhibitors etc. In certain situations, it might be advantageous that shorter courses of radiotherapy interfere less with administration dates of systemic treatment. However, all these issues require additional studies.

Going back to the combination of radiotherapy and bisphosphonates, Canadian data suggested that use of bisphosphonates has not reduced the utilization rates of palliative radiotherapy in breast cancer patients with bone metastases [39]. There was a trend of initiating bisphosphonates before delivery of palliative radiotherapy.

A Turkish randomized study (also limited to breast cancer) suggested that high-dose palliative radiotherapy was equally effective as reduced-dose irradiation when used concomitantly with zoledronic acid [40]. A second, non-randomized study (different primary tumors) confirmed this finding and suggested that bisphosphonates did not have any additive effects on pain palliation in the management of painful bone metastases with radiotherapy [41]. It is therefore unlikely that variations in systemic treatment are the major reason for the large differences in utilization of single fraction radiotherapy.

Another potential reason is financial incentives, even if not confirmed by the responses of Italian survey participants (apparently this question was not addressed in the other surveys). If reimbursement of single fraction radiotherapy is worse than that of a course of multiple fractions, it might be more attractive to prescribe a longer course. Clearly this issue depends on health care system, reimbursement rates and departmental waiting lists or patient prioritization. In Norway (all patients covered by the National Health Care system, no private radiation oncology providers), reimbursement actually was influenced in part by number of fractions. Nevertheless single fraction use was quite common [34]. Because of inadequate nationwide capacity and lower than recommended general utilization of radiotherapy (estimated need was 55% of all cancer patients as described in the National Cancer Treatment Program), waiting lists existed in all radiotherapy departments despite operating at maximum capacity. It was therefore not possible to increase the departmental revenue by prolonging palliative radiotherapy series. Any such attempt would have caused a disadvantageous increase in waiting time. The considerations discussed here provide arguments for creating reimbursement scenarios that remove incentives for prolonged fractionation regimens. However, the situation is complex because there is obvious agreement in all radiation oncology centers and communities represented in the studies shown earlier, that a general "less is more" approach is not warranted. In other words, one size (in this case single fraction radiotherapy) does not fit all patients. Recent guidelines appreciate the fact that any treatment recommendation must fit the individual need of a given patient, and that we have to choose appropriately from several fractionation options [42-44]. If there is a medical need for more intense radiotherapy despite of higher economic burden (an example of cost-effectiveness is given in [45]), any additional operating expense should also be reimbursed. For the purpose of this mini-review, we will not discuss the ability to deliver single fraction or hypofractionated stereotactic body radiotherapy to certain metastatic sites [46,47]. The basic dilemma also relates to stereotactic techniques: if certain medical indications for more intense radiotherapy exist, how can these be clearly defined and agreed on?

The question can also be rephrased: Have previous studies given us all the answers we need? One of the randomized studies was performed at the first author’s previous institution [15]. Even if no single fraction arm was included, it highlights the challenges of previous bone metastases research. We included patients with all types of solid primary tumors, different bone and extra-osseous disease extent, location in pelvis, spine, ribs and so on, and in different disease trajectories (bone metastases at first cancer diagnosis in treatment-naïve patients, heavily pre-treated patients without further systemic treatment options etc.), in other words, all those patients practicing radiation oncologists meet during a typical month. Prognosis differed but should this fact influence our choice of fractionation regimen? In the prospective randomized Dutch bone metastasis study (single fraction of 8 Gy versus 24 Gy in six fractions), 28% of the patients survived for more than 1 year [48]. In these 320 patients with better prognosis, responses were 87% after 8 Gy and 85% after 24 Gy (p=0.54). Duration of response and progression rates were similar. For all primary tumors, prognostic factors for survival were good performance status, no visceral metastases, and non-opioid analgesics intake.

**Figure 1:**
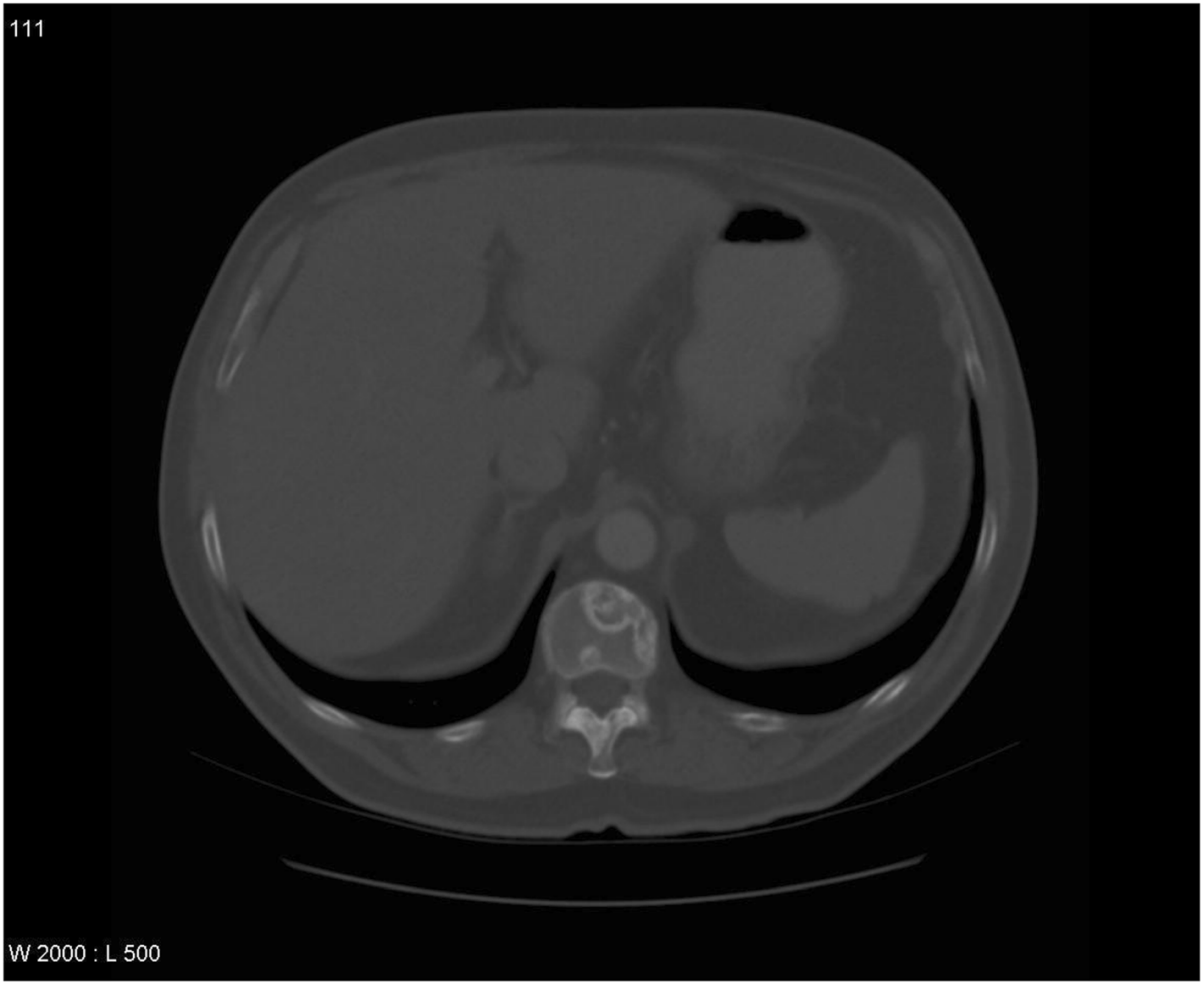
Computed tomography scan in a patient with adenocarcinoma of the prostate, multiple bone metastases and uncomplicated bone pain (bone lesions not causing neurological complaints and without a high risk of pathological fracture), no extra-osseous extension: treated with 8 Gy x1.

**Figure 2:**
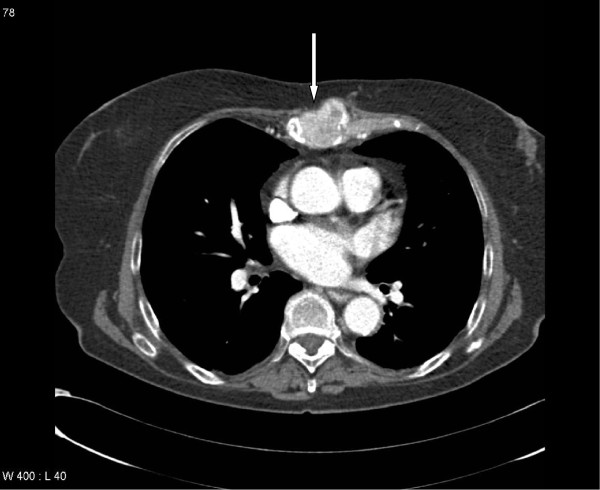
Computed tomography scan in a patient with clear cell kidney cancer and uncomplicated bone pain (bone lesions not causing neurological complaints and without a high risk of pathological fracture), extra-osseous extension present (white arrow), known lung and adrenal gland metastases: treated with 8 Gy x1.

**Figure 3:**
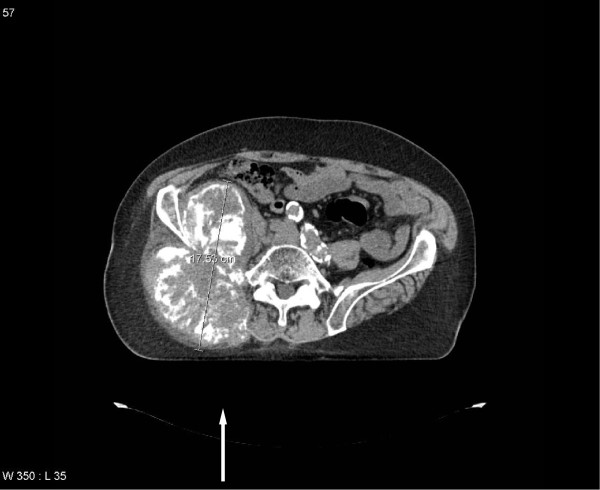
Computed tomography scan in a patient with adenocarcinoma of the lung (no EGFR mutation) and bone pain (bone lesion causing weakness of the right lower limb, initial manifestation of this lung malignancy, treatment-naïve), massive extra-osseous extension present: treated with 3 Gy x10.

**Figure 4:**
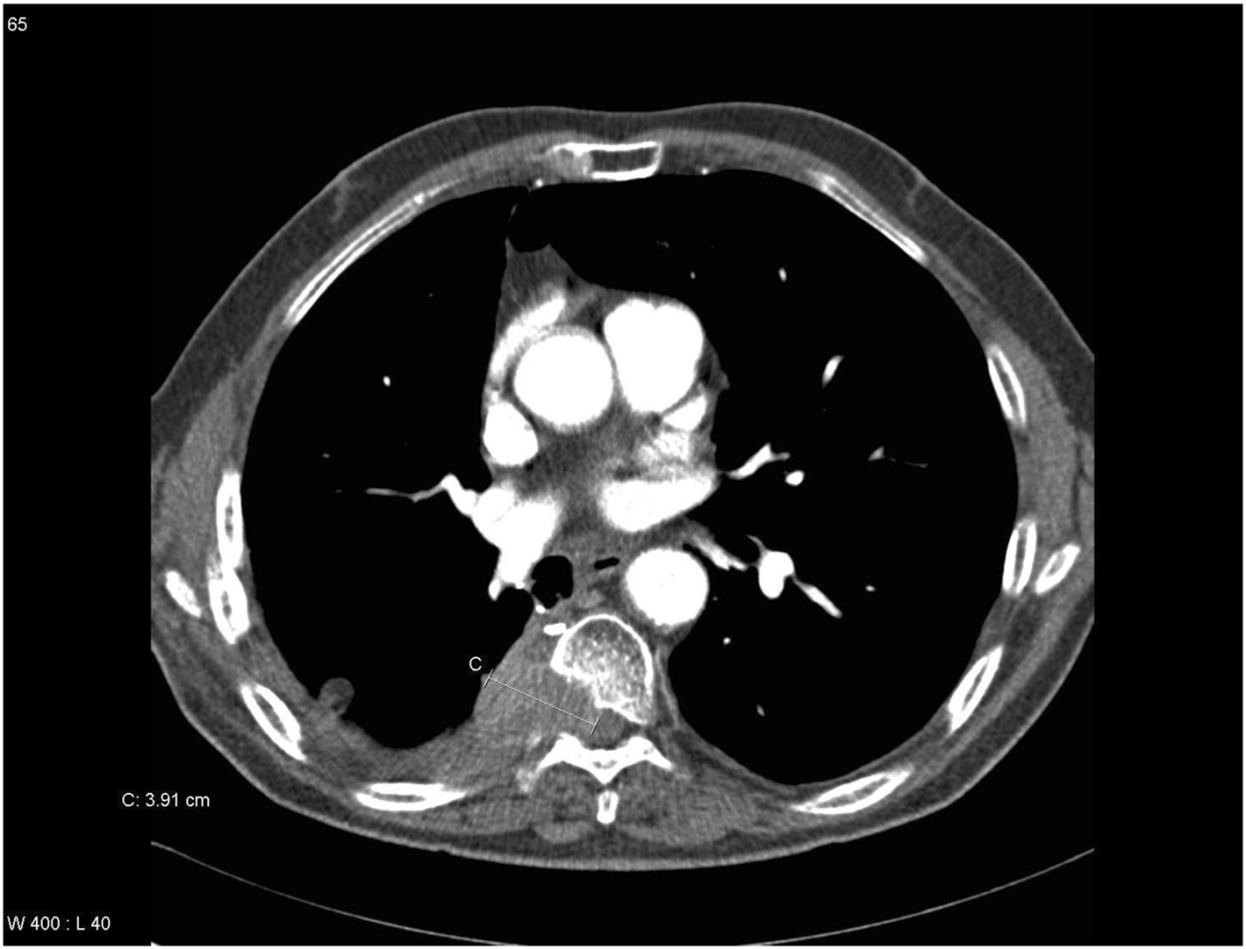
Computed tomography scan in a patient with squamous cell lung cancer (no EGFR mutation, known lung metastases) and uncomplicated bone pain (no neurological complaints), extra-osseous extension and spinal canal invasion present: treated with 3 Gy x10.

Figures 1, 2, 3 and 4 show the broad variation of local disease extent, another factor considered by many physicians. Most likely, more radiation oncologists might be reluctant to use single fraction radiotherapy in examples 3 and 4 (large volume disease and/or threat to spinal cord) especially if the patients' survival expectation is not limited to 2–3 months, they are ambulatory and in good general condition. The randomized trials have not specifically addressed patient populations with large volume disease, which consist of some patients with uncomplicated bone pain (bone lesions not causing neurological complaints and without a high risk of pathological fracture) and some patients with impending fracture or spinal cord compression. For reasons including but not limited to medico-legal issues no uniform international threshold for definition of impending spinal cord compression or increased fracture risk exists. Performing retrospective analyses of older randomized trials that identify such challenging patients is difficult and methodologically inferior to conducting trials limited to narrowly defined patient groups and including all relevant endpoints. Future trials could also shed light on other interesting questions many practitioners are struggling with: does the higher biologically effective dose of more intense regimens truly provide more extensive tumor remission and/or superior local control (remember that pain relief is not clearly related to pre-treatment or longitudinal imaging findings) and if so, do serial imaging findings eventually translate into clinically measurable benefit? At the end of the day we care for patients with limited survival expectation and complex disease states, which impair quality of life at different levels. Can we achieve the same outcome by reirradiating those patients who do not experience long-lasting benefit after their first course of radiotherapy?

We hypothesize that most clinicians already prescribing single fractions to many but not all of their patients are making careful decisions based on many clinical variables. They try to assign the right patient to the right treatment. Yet not all of these variables might be truly relevant, and future studies should also aim at development of decision aids that are just as complex as needed, become generally accepted and part of treatment guidelines. Both radiation oncology communities and bone metastasis experts should support studies providing the evidence that will allow practitioners to escape the current dilemma of often subjective treatment recommendations. It is unfortunate that comparable patients receive heterogeneous treatment even within well defined regions (for example Ontario [35]) or tightly regulated and centralized cancer care systems with small populations (for example Norway [34]). In Ontario, single fractions were used more frequently in patients who lived further from a radiotherapy facility and when the prevailing waiting time was longer [35]. How should these factors be weighted when deciding about treatment, both on an individual patient basis and a more general level (who pays for transportation and accommodation, is access to curative radiotherapy threatened by a wealth of patients receiving long-course palliative treatment)? We believe it is possible to perform additional studies that will better define which patients cannot be adequately managed with a single fraction of 8 Gy, which is considered to be the preferred radiotherapy schedule for patients with uncomplicated bone pain, and to determine the optimum regimen for these patients.

## Competing interests

The authors declare that they have no competing interests. They are affiliated with two Norwegian institutions in Bodø and Tromsø that were covered by a recent study [34]. Both institutions had prescribed single fractions of 8 Gy more often than national average but less often than the 54% of the leading institution. Their clinical practice is by no means better or more objective than yours. They are reading the seminal papers and do not find all answers they would like to get. When it comes to the title of this paper, how can they avoid to blame themselves, i.e. the doctors? Who else has responsibility for delivering the goods, i.e. treating our patients as participants of those clinical trials that give us more answers.

## Authors’ contributions

CN and AD participated in the design of the study. CN and AP performed the literature search, extracted relevant articles and drafted the manuscript. All authors read and approved the final manuscript.
